# Unravelling the druggability and immunological roles of the SOCS-family proteins

**DOI:** 10.3389/fimmu.2024.1449397

**Published:** 2024-11-29

**Authors:** Dylan M. Lynch, Beth Forrester, Thomas Webb, Alessio Ciulli

**Affiliations:** Centre for Targeted Protein Degradation, Division of Biological Chemistry and Drug Discovery, School of Life Sciences, University of Dundee, Dundee, United Kingdom

**Keywords:** SOCS proteins, E3 ligases, small molecule inhibitors, cell signalling, Cullin RING E3 ligases, targeted protein degradation, SH2 domains, phosphotyrosine (pTyr)

## Abstract

The Suppressor of Cytokine Signalling (SOCS) protein family play a critical role in cytokine signalling and regulation of the JAK/STAT pathway with functional consequences to the immune response. Members of this family are implicated in multiple different signalling cascades that drive autoimmune diseases and cancer, through their binding to phosphotyrosine modified proteins as well as ubiquitination activity as part of Cullin5 RING E3 ligases. Here we review the SOCS family members CISH and SOCS1-SOCS7, with a focus on their complex role in immunity. The interactome and signalling network of this protein family is discussed, and the intricate mechanisms through which SOCS proteins alter and manage the immune system are assessed. We offer structural insights into how SOCS proteins engage their interacting partners and native substrates at the protein-protein interaction level. We describe how this knowledge has enabled drug discovery efforts on SOCS proteins to date and propose strategies for therapeutic intervention using small molecules, either via direct inhibition or leveraging their E3 ligase activity for targeted protein degradation.

## Introduction

Cytokine signalling is a cornerstone of the immune system, mediating a plethora of complex responses to facilitate the development of immune cells. Dysregulation in cytokine signalling gives rise to a number of immune defects such as inflammation, and several immune-centred malignancies. Multiple protein families are therefore involved in maintaining cytokine signalling, often through negative feedback loops used to restrain the signalling cascade. The inhibition and knockdown of these proteins releases this blockade of negative feedback on the immune system. Therapeutic approaches to the mediation or abrogation of cytokine effect can be considered through different lenses: a *“shoot the messenger”* approach, in which cytokine ‘messengers’ can be directly bound and sequestered through targeted antibody therapy approaches (preventing their signalling and downstream effects) or alternatively, induction of Suppressor Of Cytokine Signalling (SOCS) protein expression can physically block or degrade the cytokine receptor, preventing binding of cytokines. For the purposes of this review, we will focus on the dynamic relationships of SOCS proteins and their receptor relationships, and readers are directed to other reviews present in the literature that discuss antibody therapy approaches (1–3).

SOCS proteins are inhibitors of the Janus kinase-signal transducer and activator of transcription (JAK-STAT) pathways, and operate in negative feedback regulation. Following their discovery just prior to the turn of the millennium (4–8), this family of immunological ‘brake’ proteins has been a crucial puzzle piece in understanding immunity and cell signalling, and has long frustrated medicinal chemists and structural biologists alike. A significant volume of research has been conducted on the SOCS family (9–13), and many of the key publications that illustrate their rich history are discussed herein.

The inhibitory activity of the SOCS proteins is largely accomplished through the assembly of a Cullin5-RING E3 ligase complex, with SOCS proteins acting as the substrate recognition subunits. The C-terminal SH2 domain contains a phosphotyrosine (pY) binding site which imparts recognition for phosphorylated native substrates, with the pY binding site located in the βE -> βF loop region (14, 15). This multisubunit protein complex catalyses the ubiquitination of the signalling components, followed by subsequent proteasomal degradation. The conserved SOCS-Box domain at the proteins C-terminus is responsible for the formation of this E3 ligase complex and is present across all eight SOCS family members: SOCS1-7, and Cytokine-Inducible SH2 domain containing protein (CISH). The *ca.* 40 residue SOCS-Box sequence recruits the adaptor proteins Elongin B and C (EloBC), and the Cullin5 (Cul5) scaffold protein (16). These Cullin-RING-ligases (CRLs) are the largest class of E3 ligases, and are of significant research interest in cell signalling, immunology, and more recently targeted protein degradation (17, 18). The C-terminal domain of the Cullin scaffold binds an activated E2-ubiquitin conjugate while the N-terminal domain locates the substrate. The Cullin scaffold is further activated by neddylation, the covalent modification of Cullin with the ubiquitin-like protein Nedd8, which dramatically increases ubiquitination activity. The post-translation modification with Nedd8 occurs on a conserved lysine residue of Cullin close to the E2-ubiquitin docking site, and results in the release of the E2 enzyme from the complex core, facilitating easier access to a substrate lysine residue via conformational bias and multivalent induced protein-protein interactions. (19).

There is still much to elucidate about how SOCS proteins mediate the intricate signalling pathways of the immune system, and beyond. While structural information is available for many of the family members, drug discovery efforts have been slow, relying largely on virtual screens or based on established treatments for diabetes and other diseases. This is largely because the orthosteric pY binding site is shallow and highly polar, making it difficult to target with small molecules. **Table 1** summarises the various mechanisms of the SOCS proteins, and their key interactions with cytokines and receptor targets. In many cases, an interaction is well established, but the precise mechanism of inhibition by SOCS proteins is not yet clarified. Protein Data Bank (PDB) entries which highlight the described native substrate interactions exist for SOCS1, SOCS2, SOCS3, and SOCS6 (PDB codes in **Supplementary Table S1**, of the Supp. Information). Structural information can accelerate the drug design process by providing a detailed understanding of the interaction landscape surrounding orthosteric binding pockets or illuminating non-orthosteric pockets and protein-protein interfaces which may be targeted with small molecules for therapeutic effect. This data can be leveraged for the design and elaboration of small molecule ligands for such sites and is particularly valuable for multimeric complexes where the interface of two proteins can be explored.

**Table 1:** Mechanisms, key interactions, and drug design efforts of SOCS family members.

Protein	Mechanism	Key Interactions	Drug Design Efforts
CISH	Competitive Binding Degradation/Re-routing	IL-12 IL-3, 4, 15 GM-CSF PLC-γ	N/A
SOCS1	JAK Inhibition Competitive Binding Degradation	IFN-α/β/γ IL-2, 4, 7, 12, 15, 21 IL-2, 15 IRS1 and IRS2	Virtual screening hits, SH2 domain and SOCS Box targeted
SOCS2	JAK Inhibition Degradation	IL-2, 3, 4, 15 GM-CSF GHR, EpoR	Covalent inhibitors, SH2 domain targeted
SOCS3	JAK Inhibition Degradation	IL-6, 12, 23 G-CSF IRS1 and IRS2	Virtual screening hits, SH2 domain targeted
SOCS4	Unknown	EGFR	Structural information published
SOCS5	Competitive Binding	IL-4/IL-4R	N/A
SOCS6	Unknown	FLT3 receptor IRS1, 2, 4	N/A
SOCS7	Unknown	IGF-1	Used in degraders

**Figure 1:**
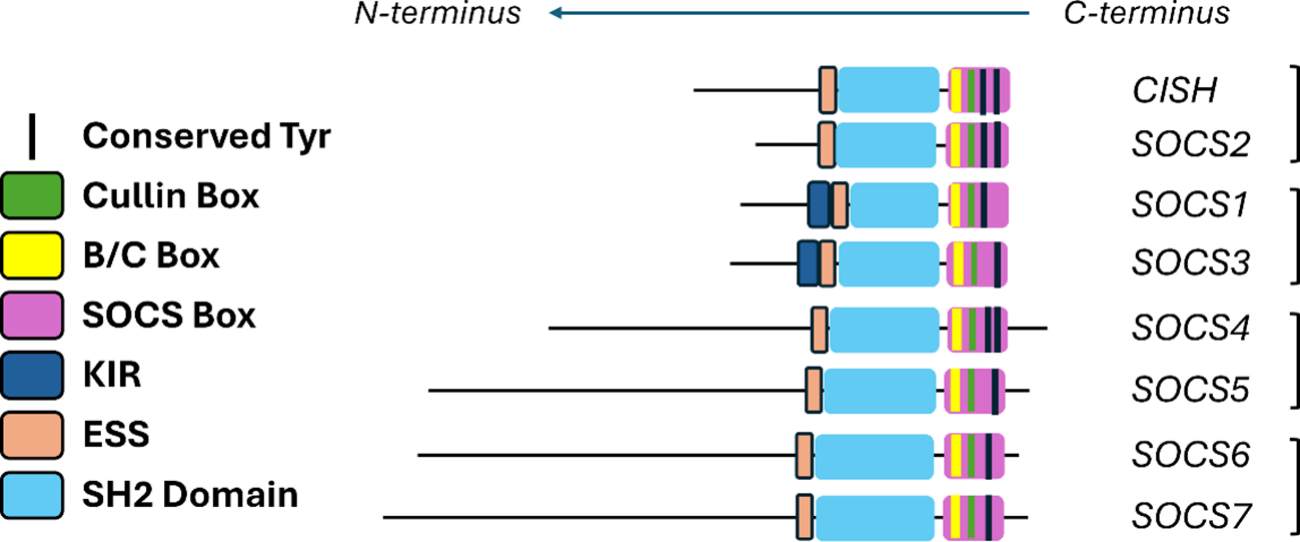
Summary of the various domains of the SOCS protein family. Extended SH2 sequence (ESS) and kinase inhibitory region (KIR) are shown in addition to the SH2 domains and the Box regions.

The similarities and differences in SOCS protein functionality can be understood in part through an analysis of their structural and functional domains. **Figure 1** highlights the structural domains of the SOCS family members. Similarities in structural domains can be attributed to a high degree of sequence consensus in structurally conserved regions. Whilst all SOCS family members contain an SH2 domain and a C-terminal SOCS, B/C and Cullin box, only SOCS1 and SOCS3 contain a KIR which is otherwise a disordered region at the N-terminus in the case of the remaining SOCS family members. These structural features are clearly implicated in SOCS protein functionality and correlate well with the elucidated mechanisms summarised in **Table 1** (20, 21).

As we discuss the various SOCS family members, a brief outlook on structural information is provided. To investigate the extent to which SOCS family members are currently structurally enabled, we sought to analyse existing PDB entries and extrapolate the number of entries per family member and the structure determination methods employed. The results of this analysis are summarised in **Supplementary Table S1** (**Supporting Information**), showing the full list of SOCS family PDB entries including PDB codes, structure determination technique, and important features. A total of 22 PDB entries are present for SOCS1-6, and no existing entries for SOCS7 or CISH. Most structures were determined by X-ray crystallography, with the remainder having been determined by in-solution protein-observed NMR spectroscopy. Two entries exist for SOCS1, with one entry for each of SOCS4, SOCS5 and SOCS6 (21–24). All these entries are X-ray crystal structures, except for SOCS5 whose structure was determined by NMR.

CISH and SOCS1-3 are some of the most attractive and closely studied targets in immune signalling and influence myeloid cell activation and lymphocyte polarisation through their effect on certain essential cytokines, such as IL-6 and interferon-γ (IFN-γ) (25). While the SOCS proteins are intimately involved in the regulation of the immune system, their conserved SH2 domain and highly polar protein-protein interaction (PPI) surfaces have often been termed ‘undruggable’; the generation of inhibitors and the study of their effects has remained challenging. In this review, we discuss in detail the immunological roles of each SOCS protein and assess the downstream effects of silencing studies, and ultimately how this can inform drug discovery efforts.

## CISH

### CISH is an independent regulator of the immune system, and an inflammatory ‘brake’ protein

Cytokine-Inducible SH2-containing protein (CISH) is involved in a wide array of disease pathways and so there is substantial interest in developing probes for this protein in order to further investigate its role as an immunological regulator. Recent studies have shown CISH as not only a STAT-dependent regulator, but as an independent regulator of the immune response (25, 26). CISH appears to serve a dual function with studies demonstrating its anti-tumour role as well as function as an inflammatory brake protein. CISH, along with other members of the SOCS family, can be thought of as “STAT-induced STAT inhibitors” due to their feedback activity.

CISH signals via the JAK-STAT pathway in response to extracellular ligand binding. CISH expression can be induced by multiple interleukins, interferons, and other molecules such as growth hormone (GH), granulocyte-macrophage colony-stimulating factor (GM-CSF), and erythropoietin (EPO). Stimulation of the receptor also activates signal transducer and activator of transcription 5 (STAT5), which is essential for CISH expression. This was demonstrated by a STAT5A/B-knockout mouse where there was no detectable CISH expression (27). STAT5A and B are predominantly involved in growth hormone and prolactin signalling. The growth hormone and prolactin receptors share a similar structure and upon cytokine binding to the extracellular domain, intracellular signalling cascades are triggered. The JAKs trans-phosphorylate each other leading to phosphorylation of the tyrosine tails. Phosphorylated tyrosine (pY) then acts as docking sites for STATs, which bind via their SH2 domains. Once phosphorylated, STATs are released from the phosphotyrosine residues due to a conformational change and homo- or heterodimerize with another activated STAT - these dimerized STATs translocate to the nucleus and bind to cis-regulatory sequences (28). This signalling cascade can lead to the expression of multiple proteins but importantly CISH, and other SOCS proteins that signal via STAT5. The resulting proteins expressed is dependent on the cytokine or signalling molecule which bound to the receptor and initiated the cascade (26).

The ways in which CISH feedback inhibits the pathway is highly regulated. CISH can bind to a phosphorylated site to prevent downstream phosphorylation or outcompete the STATs directly preventing them from entering the nucleus and inducing gene expression. Once bound to the phosphorylated receptor, the remaining components of the Cullin5 E3 ligase are recruited via their SOCS-box and subsequent ubiquitination and proteasomal degradation of the receptor occurs (29).

**Figure 2:**
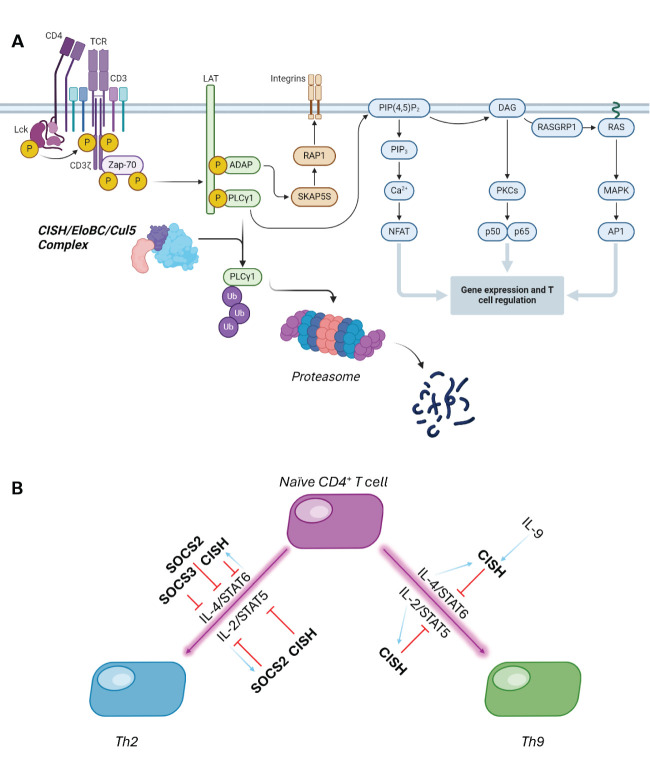
**(A)** CISH can form an E3 ligase complex with Elongin B/C and Cullin5, which catalyses the polyubiquitination and subsequent proteasomal degradation of PLC- γ. **(B)** CISH and SOCS2 work in tandem to control cytokine-mediated differentiation of T cells. Purple lines show stimulated differentiation, with red showing SOCS-dependent suppression, and light blue arrows indicating SOCS protein induction in this cascade (31).

Evidence of CISH role as an inflammatory regulator was shown in a study using CISH^-/-^ mice which had developed an increase in airway inflammation and pulmonary disease. These conditions are often indicative of excessive IL-13 and IL-9 production. In CISH deficient mice, T cell differentiation into Th2 and Th9 cells is enhanced by IL-4 mediated STAT6 activation (30). This STAT can be activated because there is no CISH to out compete it, but in the absence of CISH, there is over production of IL-13 and IL-9, both of which promote an allergic inflammation response and exert an effect on airway epithelium. CISH is crucial to control the magnitude of this signalling response pathway – the balance between suppression of differentiation and SOCS-family stimulation is shown in **Figure 2B** (32).

CISH is also known for its inhibitory effect on adaptive immunity and T cell activation. T cells are required for targeting infected cells for degradation as well as activating other immune responders, like B cells. For example, CISH binds to EloBC-Cul5 and recruits PLC-γ as substrate, catalysing ubiquitination and substrate degradation. In the absence of CISH, PLC-γ is able to convert PIP3 into IP2 and DAG which increases calcium flux as well as activate several enzymes. Both PLC-γ driven events are crucial to activate transcription of key T cell regulators and hence in the presence of CISH there is significant reduction in the transcription factors vital to T cell activation, reducing T cell differentiation and function (**Figure 2**) (33).

CISH have important functions in infection-mediated immune cells such as CD4^+^ and CD8^+^ (26). One of the difficulties in studying the SOCS protein arises because they are not traditionally constitutively expressed (34). Their respective cytokine receptor must be expressed, stimulated, and (in some cases) dimerized before there is downstream expression of the SOCS proteins. The regulation of cytokines such as interleukins is therefore responsible for the abundance of SOCS proteins. CISH is dependent primarily on the STAT5 pathway being activated which can be achieved through IL-2R stimulation. ILs primary role is to modulate differentiation, growth, and activation of multiple immune pathways (35). They are naturally produced by leukocytes, lymphocytes, monocytes, and other immune cells and are secreted rapidly in response to a stimulus such as an infection. ILs stimulate a signalling pathway which results in further IL expression, as well as other genes, which keep feeding back to boost the intended response (36).

Upon IL-2 stimulation of the JAK3/STAT5 pathway and subsequently increasing the expression of CISH, the levels of pSTAT5 decreases. CISH feeds back to limit the response of this pathway, thus affecting the magnitude of T cell related genes being transcribed and translated. IL-2 has a primary role in T cell proliferation and differentiation as well as activating lymphocytes and macrophages (37). A study investigating the relationship between phosphorylated STAT5 and T cell proliferation showed that IL-2 receptor stimulation increased the T cell proliferation amount (26). Upon JAK3/STAT5 inhibition significant suppression of T cell proliferation was observed, mimicking the effect that CISH would have inhibiting STAT5 phosphorylation by competing for the phosphotyrosine docking sites (36).

### CISH is implicated in dendritic cell-mediated CTL activation, susceptibility to infection, and combating solid tumours

Li et al. outlined that the function of CISH likely varies depending on cell-type and other factors; CISH can also activate TCR signalling and enhance T-cell populations in transgenic mice models (38). Furthermore, Khor et al. reported that mutations in the human *CISH* gene is associated with susceptibility to a number of diseases driven by pathogenic infection (39). While the mechanism of this was initially unclear, Miah et al. observed that CISH expression plays a large role in Dendritic Cells (DC)-mediated CTL activation. Furthermore, they discovered via gene silencing experiments that CISH impairs the proliferation of DC precursor cells initially, and instead triggers their differentiation into type 1 polarized DCs, which are essential for the innate immune response in pathogen infection. Miah et al. found that while CISH is not expressed in bone marrow (BM) cells, a significant upregulation is seen in later DC development. This upregulation is essential in the development of potent type 1 DCs, which are crucial in the activation of CD8^+^ T-cells. Additionally, CISH knockdown models (CISH^KD^) produced DCs which were significantly less effective at inducing antigen-specific CTLs in immunised mice when compared to the wild-type, indicating that high expression levels of CISH throughout DC development is essential (40).

In a publication from Palmer et al. CRISPR/Cas9 knockouts of CISH in tumour-infiltrating lymphocytes (TILs) further illuminated the role of CISH and showed that its knockdown conferred the ability to combat solid tumours (41). In their account, the authors describe a crucial role of CISH in regulating the reactivity of human T cell function to neoantigens. Upon knockout experiments, they observed increased TIL function and neoantigen recognition. Furthermore, they identified CISH^-/-^ as a method to increase the vulnerability of tumours to a type of cancer immunotherapy referred to as checkpoint inhibition, using *in vivo* models; this later formed the basis of a clinical trial for treating certain gastrointestinal cancers using CISH^-/-^ TIL (41). The authors concluded that the genetic disruption of CISH function offers a novel therapeutic approach to cancer, due to the negative regulation that CISH exerts on human T cell function. Zhu et al. then further validated these findings in the same year, by using a CISH^-/-^ in natural killer (NK) cells. This study identified that the deletion of CISH yielded improved anti-tumour activity, and that acute myelogenous leukaemia (AML) could be treated more effectively with CISH^-/-^ NK cells, and that these cells displayed increased persistence (42).

A recent account from Lakkavaram et al. framed CISH^-/-^ in the context of malaria infection. Their account studied the impact on cytokine and blood cell parameters in CISH^-/-^ mice infected with *Plasmodium berghei (* 43 *).* The authors identified that knockout mice maintained their haemoglobin levels and peripheral blood cell counts compared to the wild-type CISH mice. However, this depletion of CISH did not alter the outcome of infection, with both CISH^-/-^ and wild-type mice displaying similar parasitic load and cytokine responses (43).

In terms of immunological targets, CISH continues to enjoy a privileged role as a disease target. In 2023, the European Patent Office granted a foundational patent to ONK therapeutics for the use of CISH knockout NK cells in oncological therapies for cell immunotherapy. This builds on previous findings that ablation of CISH improved the NK cells response to growth factors, and drastically improved the cell fitness and ability to tackle tumour cells (42, 44, 45).

The promotion of T cell antitumour activity via disruption and deletion of CISH provides an exciting therapeutic concept. CISH inhibitors or CISH degraders e.g. homo-PROTACs could offer a new avenue in the development of cancer therapies; in the latter case essentially offering a chemically-induced pharmacological equivalent to a CISH knock-down, which could serve as an orthogonal approach to the work of ONK therapeutics discussed above.

## SOCS1

### SOCS1 regulates the JAK-STAT pathway through inhibition and proteasomal degradation

All SOCS proteins contain two conserved regions, the SH2 domain and C-terminal SOCS box domain. SH2 domains poise the SOCS proteins to bind phosphorylated residues on the receptor whilst the SOCS box acts as a substrate recognition receptor to recruit the components of an E3 ligase and mediate ubiquitination of the receptor (31). SOCS1, as well as SOCS3, house a kinase inhibitory region (KIR) which can block JAK kinase function even in the absence of SOCS box. SOCS1 inhibits its respective JAK-STAT signalling pathway through multiple modes, and is described as the most potent of its family due to its additional inhibitory feature facilitated through the KIR. This region targets the JAK binding pocket with high specificity and prevents the phosphorylation of the JAKs, blocking downstream signalling of the pathway (22).

SOCS1’s main mechanism of action in regulating the JAK-STAT pathway is through its KIR. SOCS1 has 100x lower affinity for recruiting the E3 components with respect to the rest of the SOCS family proteins (20). This is due to an atypical binding sequence in the SOCS box which lacks an essential proline; significant homology with the other SOCS proteins in the binding regions remains, with this key interaction missing (22). Hence, while SOCS1 can recruit and form an E3 ligase complex, this is not its predominant form of regulating the JAK-STAT signalling pathway. SOCS1 can also bind to phosphorylated sites on the receptor, thus blocking the phosphorylation and dimerization of STATs. Unlike other JAK-STAT pathways that SOCS feedback on, SOCS1 does not require receptor activation by cytokines before inhibiting the pathway. This is due to the KIR being able to target unphosphorylated site on the JAKs, in particular JAK1, JAK2, and TYK2 (22).

There are an abundance of interleukins and interferons which induce the expression of SOCS1. Which cytokine activates the receptor determines the different combinations of JAKs and STATs that are recruited. The pathway from receptor stimulation to expression of SOCS1 and other essential genes is highly regulated by multiple proteins: protein inhibitors of activated STAT (PIAS), protein tyrosine phosphatases (PTPs), and other SOCS proteins themselves. PIAS works through SUMOylation of the STATs and JAKs, whilst PTP dephosphorylates tyrosine residues thereby obviating the docking site for STATs to bind and activate (46). These three protein groups all rely on altering the conformation of the receptor and therefore interfering with the normal binding pathways.

STAT1/2/4/5/6 can be activated through phosphorylation resulting in the expression of SOCS1. The plethora of STATs and cytokines with the potential to trigger SOCS1 expression, can result in co-expression of multiple other genes and proteins. Regulation of the interleukins and interferons which induce SOCS1 expression are produced and released from an array of immune cells such as T cells, NK cells, B cells, and macrophages (47).

A fundamental role of SOCS1 is its role as a tumour suppressor (22). SOCS1 silencing has been identified across multiple tumour types; this is due to frequent epigenetic and micro-RNA mediated suppression of its gene expression (48). SOCS1 can reduce tumour prosperity through promotion of p53 transcriptional activity and inhibiting p21 oncogenic functions (49). The SOCS1 SH2 domain can interact with the N-terminal TAD2 domain of p53 at DNA damage loci resulting in the phosphorylation and subsequent activation of p53 (50). This phosphorylation event increases the activity of p53 as a transcription factor, translating genes into proteins which are crucial in preventing the formation of tumours. (51).

IFN-γ is one of the most well characterized targets for SOCS1 action, although a report by Liau et al. observed no binding between SOCS1 and peptide fragments of IFN-γ via isothermal titration calorimetry (ITC) (22). This is achieved through SOCS1’s prevention of T_reg_ cells producing IFN-γ by suppression of STAT1. The downstream affects are a lack of essential genes that are required to convert T_reg_ cells into effector T cells. There lies controversy in this auto-suppression by which other signalling cascades triggered by IFN-γ can positively regulate STAT1 transcription and thus allow regulatory sequence binding and expression of specific proteins (52).

### SOCS1 acts as the most potent SOCS family member, and is essential in insulin signalling

SOCS1 is essential in controlling inflammation and maintaining immune homeostasis (53). New substrates of SOCS1 are still being discovered, and while targeting this protein has potential in many immune disorders, not all effects of SOCS1 are advantageous in the context of autoimmune diseases. SOCS1 induces degradation of essential components of insulin signalling via the conserved SOCS Box domain – one account from Dumpati et al. in 2018 investigated potential binders of SOCS1 through the lens of therapeutics for type 2 diabetes mellitus (T2DM) (54). SOCS1 induces proteasomal degradation of IRS1 and IRS2, which in turn results in insulin resistance in T2DM. Using computational modelling of large virtual databases, this account identified lead structures for potential SOCS1 binders, based on 3-pyridinol, xanthine, and others. Furthermore, the authors indicated glyclopyramide and gilbenclamide as drugs with partial affinity for SOCS1, and good drug-like properties. Ahmed et al. had earlier identified that SOCS1 deficiency could be potentially treated with SOCS1 mimetics, but this approach was impaired due to the mimetic peptides lacking the SOCS Box sequence. Ahmed and co-workers also uncovered that a peptide corresponding to the activation loop of JAK2, pJAK2(1001–1013), could act as a SOCS1 antagonist and was able to enhance both innate and adaptive immune responses to several viral pathogens (55).

SOCS1 is a primary regulator of IFN-γ and multiple other cytokines, and is generally regarded as the most potent of the SOCS proteins; furthermore, SOCS1^-/-^ is lethal. Mouse models with genetic deletion of SOCS1 die from general inflammation after 2 weeks (22, 56). Rescue from neonatal death can be accomplished by also knocking out IFN-γ, however these mice still suffer from reduced viability compared to the wild-type (22). Liau et al. rationalised the design of a small molecule mimetic of the kinase inhibitory region (KIR) of SOCS1 by solving two X-ray crystallography structures of SOCS1 bound to Elongin B/C adapter complex, and separately the JAK1 kinase domain, which would enable the development of novel JAK inhibitors. Although active signalling by IFN-γ is essential for effective immunotherapy within an oncology context, small-molecule inhibition of SOCS1 should dramatically increase the efficacy of these therapies (22). Recent work from Babon and co-workers provides basis for the structure-guided drug design of novel SOCS1 inhibitors (22).

SOCS1^KD^ and SOCS1^KO^ models have been achieved through various methods, with important physiological consequences. Hildebrand et al. achieved potent silencing of SOCS1 using lipid nanoparticle-enclosed siRNA (L-siRNA), which protected the construct from early degradation and abrogated several toxic side effects. Through a targeted release of the siRNA upon translocation to the cytoplasm, the termination of NF-κB signalling was obviated due to its dependence on SOCS1 mediation. The authors illuminated this inhibition of SOCS1 as a method to increase peptide vaccination immunogenicity, by increasing TLR4-adjuvant stimulated monocyte activation and improving T-cell responses. SOCS1 inhibition in this case disrupts the negative feedback loop in antigen presenting cells (APCs), permitting the development of more potent vaccines for infectious diseases, and strengthen vaccination against oncogenic viruses (57). Luo et al. further investigated the long-term effects of SOCS1^KO^ in skin-resident CD4^+^ cells, and found that in the context of a protracted contact-allergic reaction, autonomous skin inflammation was evident and featured aspects of a skin lymphoma, Sézary Syndrome (mycosis fungoides) (58). This coincides with the recent genomic analysis of mycosis fungoides which identified SOCS1 and one of the frequently deleted tumour suppressors in this disease; a one copy deletion of SOCS1 has been identified in lesions exhibited in early stage Sézary syndrome (58, 59).

SOCS1 plays an invaluable role in balancing inflammatory cytokine signalling, and is thoroughly involved in maintaining the equilibrium between beneficial and disadvantageous biological effects. It plays a critical role in the regulation of IFN-γ signalling cascades, and has many complex roles involving T cell activation, JAK/STAT signalling, and the inhibition of insulin signalling. Further studies of the cross-talk between SOCS1 and the SOCS family members is needed to cement SOCS1 as a useful therapeutic target for autoimmune diseases and cancers.

## SOCS2

### SOCS2, an exciting therapeutic target, is indispensable in regulating inflammatory responses

SOCS2’s functionality as a suppressor of cytokine signalling lies predominantly in its ability to outcompete STAT binding to phosphorylated receptors, as well as via its E3 ligase activity. A well characterised substrate of SOCS2 is the growth hormone receptor (GHR). Activation of GHR triggers SOCS2s involvement in the negative feedback loop with GH. Greenhalgh et al. reported on gigantism associated with SOCS2^-/-^ in mice, reporting a 30-50% increase in mature body size for SOCS2^-/-^ (60, 61). SOCS2 therefore acts as a molecular brake on many inflammatory pathways to control the magnitude of an immune response, and protect the cell from damage.

**Figure 3:**
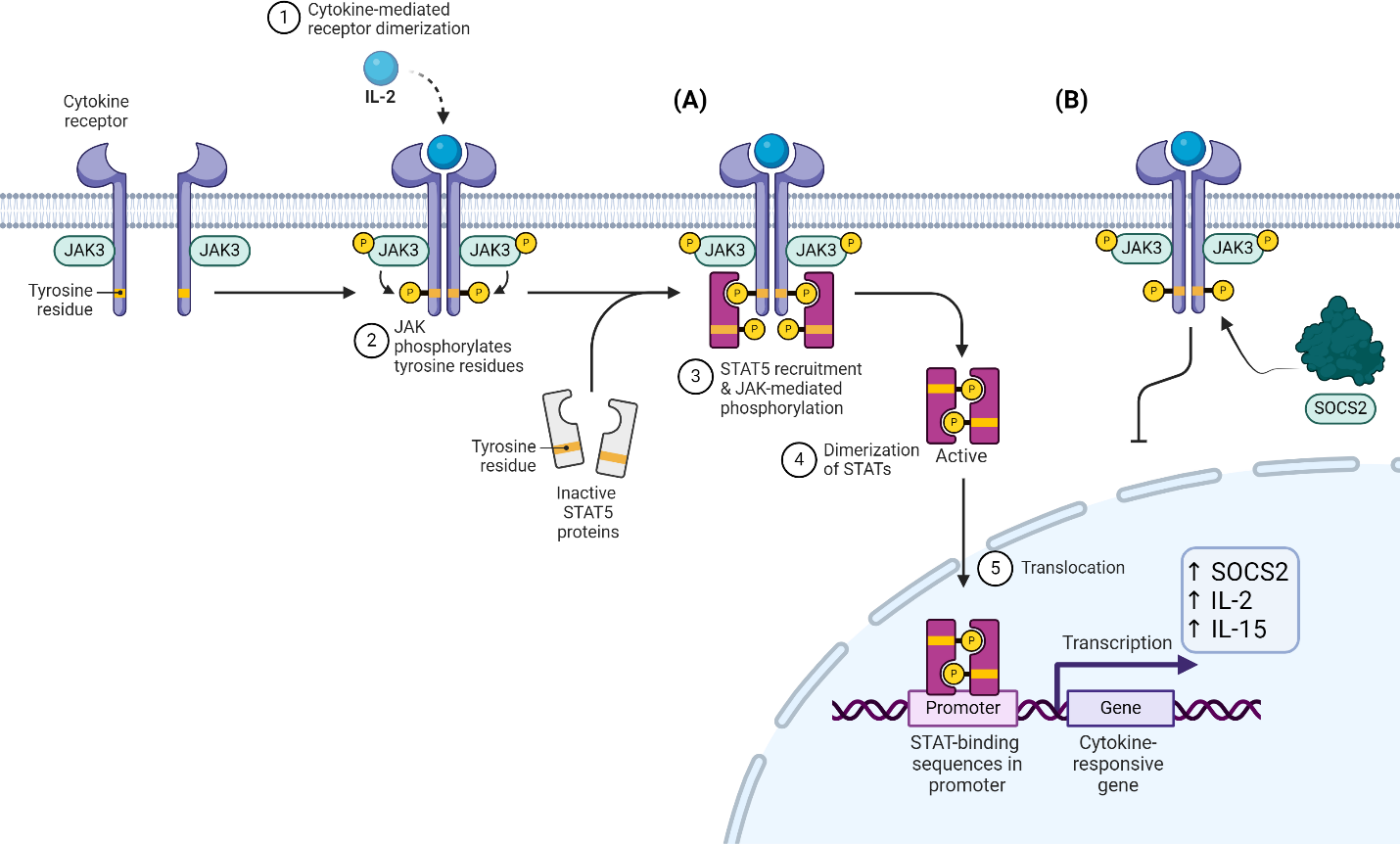
**(A)** IL-2 stimulated JAK-dependent phosphorylation and dimerization of STAT5. **(B)** SOCS2 competes with STAT5 for pY residues, inhibiting this cascade.

SOCS2 expression is induced by the activation of the JAK3/STAT5 pathway, and to a lesser extent STAT6. Cytokines which stimulate this pathway are predominantly IL-2, 15, 3 and IL-4 through STAT6. Once the STATs are phosphorylated, they dimerize and translocate to the nucleus to induce expression of multiple cytokine-related genes, including SOCS2 (**Figure 3A**). SOCS2, like others in its family, act as feedback inhibitors to compete with STAT5 for the phosphorylated tyrosine residues (**Figure 3B**). This was demonstrated in K562 cells where SOCS2 was shown to interact directly with a peptide fragment of the phosphorylated GHR, a native substrate for SOCS2. Pulldown assays were performed to show that SOCS2 could be ‘fished’ out of whole cell lysates by binding to this GHR peptide. It was also shown that this interaction was abolished if SOCS2 was pre-incubated with an inhibitor that blocks the SH2 binding domain (14). This new data highlighted how crucial this binding pocket is in recognising and binding to the phosphorylated sites to slow down the cytokine signalling, and if SOCS2 was inhibited, the signalling pathway would proceed as normal.

IL-2 signalling results in the downstream translation of genes vital to the terminal differentiation of T cells into short-lived effector cells. This signalling cascade also expresses more IL-2 protein to further amplify the cytokine effect. IL-2 drives an increase in cell killing activity of multiple immune cells such as NK and cytotoxic T cells. The regulation of these cytokines is tightly controlled due to their potential in mounting a large immune response which could lead to a cytokine storm. One key checkpoint before IL-2 is released is an interaction dependency between a T cell receptor and a human leucocyte antigen (HLA)-peptide complex. This main role of this complex is to bind antigenic fragments and display them for recognition by lymphocytes specific to them (62). HLA’s help the immune system differentiate between self-proteins and foreign proteins. There is a positive feedback loop involving the production of IL-2 by activated T-cells, which further promotes more T cells, and IL-2. SOCS2 therein acts as natural brake on the IL-2 pathway by inhibition of the receptor through blocking STATs phosphorylation potential.

IL-15 is produced by monocytes and is an activator of T cells. The IL-15 signalling pathway regulates the activation and proliferation of T cells and NK cells. A vital function of IL-15 is that they maintain memory T cells even once the antigen has been cleared (63). IL-15 signals through STAT5 and interestingly the IL-15 receptor mRNA levels can be increased in response to IL-2 release in T cells. An altruistic relationship to maximise cytokine effect (64). This signalling pathway also leads to the production of other cancer-fighting and anti-inflammatory related proteins, such as TNFα which functions by regulating inflammatory cytokine production. SOCS2’s role as an inflammatory regulator has been seen in an inflammatory bowel disease study (65). Deletion of SOCS2 displayed activation of pro-inflammatory signalling pathways whereas in WT SOCS^+/+^ mice, SOCS2 showed anti-inflammatory activity through its ability to ubiquitinate cytokine receptors.

The therapeutic interest around SOCS2 is growing rapidly due to its regulatory involvement in multiple immune response pathways (31). Following the recent publication of a SOCS2 inhibitor, there is renewed potential to probe SOCS2’s function in immune cells and unravel more insight into its role as an E3 ligase.

### Knockout effects, drug design targeting SOCS2, and its E3 ligase complex

SOCS2 is one of the only SOCS family members for which significant drug design efforts have been published. Structure-guided drug design has primarily targeted the SH2 domain of the SOCS2-Elongin B/C complex, with the aim of inhibiting the activity of this complex, and possibly utilising its E3 ligase activity for targeted protein degradation.

Monti-Rocha et al. studied the role of SOCS2 in liver injury after acetaminophen (APAP) overdose. SOCS2 plays an important role in controlling growth factor signalling and has been implicated in cellular processes in the liver. In this account, SOCS2^-/-^ hepatocytes under APAP overdose conditions produced significantly more phosphorylated NF-κB and reactive oxygen species (ROS) than the wild-type, and were more sensitive to cell death in the presence of other cytokines such as IL-6. SOCS^-/-^ mice displayed significantly more neutrophil recruitment and necrosis, with elevated proinflammatory cytokines and a serious reduction in anti-inflammatory cytokines such as transforming growth factor-β (TGF-β). The authors concluded from these knockout studies that SOCS2 plays a key role in balancing both inflammatory mechanisms and pro-oxidative pathways, and is implicated in protecting the liver during overdose (66).

In terms of ligand development for SOCS2, efforts have concentrated on drugging the SH2 domain. Since SOCS2 binds phosphotyrosine (pY) residues, medicinal chemistry efforts have focused on building phosphopeptides and expanding into structure-guided drug design. Kung et al. provided structural insights into how SOCS2 engages and recognises substrates by investigating the interaction with small peptides based on GHR and EpoR epitope sequences (67). These short sequences, based around the GHR phosphotyrosine site at pY595, and EpoR at pY426, enabled the authors to obtain crystal structures and observe conformational changes exhibited by SOCS2 upon peptide binding. Furthermore, this account highlights the effects of single nucleotide polymorphisms (SNPs) in SOCS2, which have been implicated in lung, breast, and pancreatic tumours (68). One key finding was that an R96C mutation obviates substrate binding by SOCS2, impairing its ability to effectively act in balancing cytokine signalling and immune responses. SOCS2 is clearly an attractive therapeutic target for immune system dysregulation, and the work of Kung et al. provides structural and biophysical insight for further drug development.

**Figure 4:**
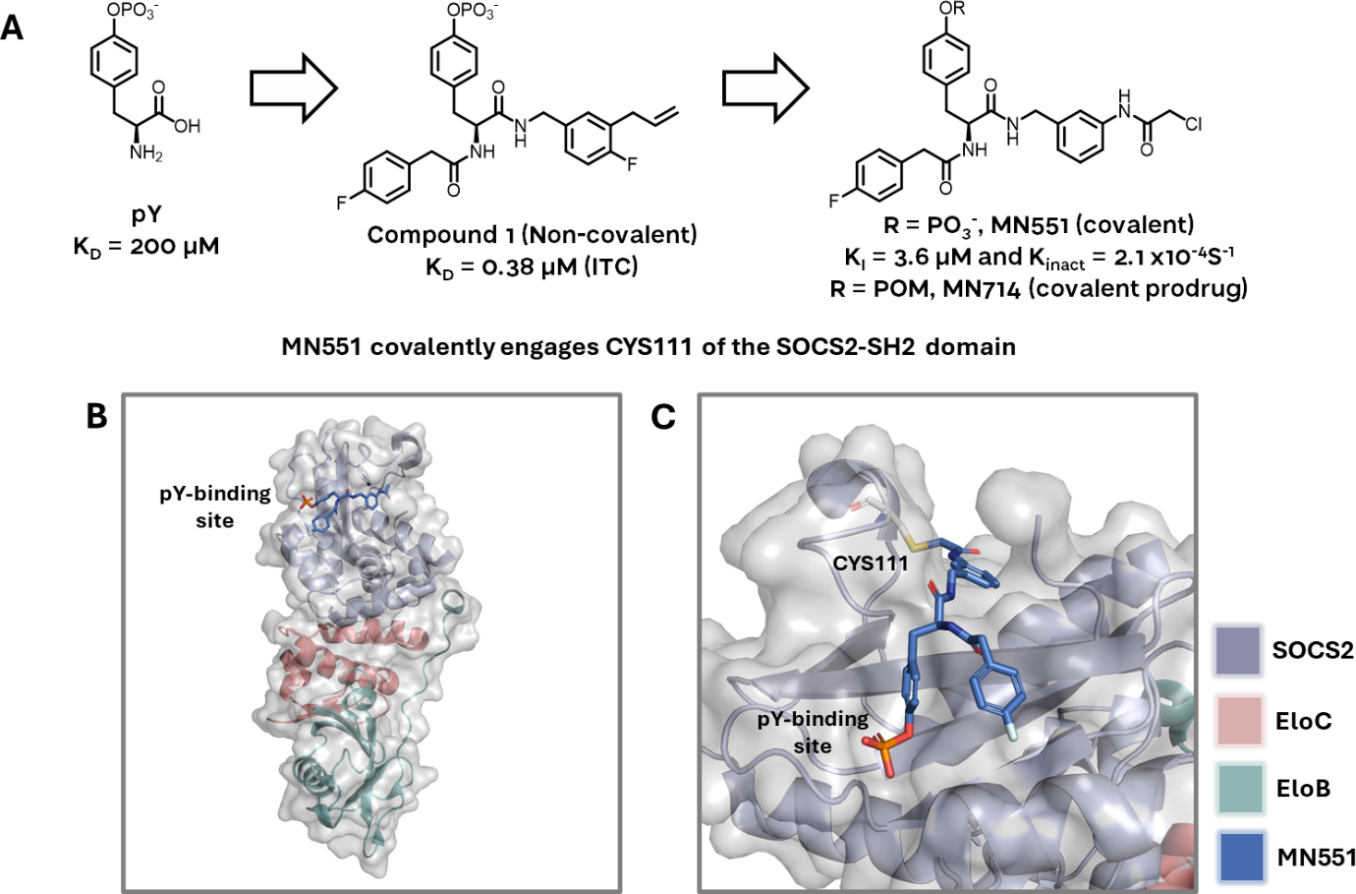
**(A)** The SOCS2-SH2 domain binder MN551 is grown from a phosphotyrosine fragment. Non-covalent compound 1 (middle) was used as the basis for structure-guided design of the electrophilic warhead. Prodrug MN714 was developed to enhance cell permeability and enable cellular studies. **(B)** MN551 covalently engages CYS111 while occupying the pY binding site of SOCS2, adapted from PDB 7ZLM. **(C)** Close-up of CYS111 engagement by MN551, adapted from PDB 7ZLM.

Building on from this work, Ramachandran and Makukhin et al. developed new small-molecule ligands for the SH2 domain via structure-guided fragment-based design (**Figure 4**). Beginning from pY as the starting anchor fragment, the authors developed a potent covalent inhibitor of SOCS2 (14). The authors first grew out of phosphotyrosine as a highly ligand-efficient fragment achieving a potent non-covalent inhibitor (compound 1 K_D_ = 0.38 μM by ITC). This was further derivatised via addition of a chloro-acetamide moiety to give MN551, which covalently binds to Cys111 located adjacent to the pY binding site of the SH2 domain (**Figure 4**). The compound was fully characterized biophysically with recombinant proteins for covalent binding, and addition of a cell-cleavable phosphate pivaloyloxymethyl (POM) protecting group enabled cell permeability via a prodrug strategy. The authors proposed SOCS2 inhibitors as attractive chemical probes for studying SOCS2 biology by assessing the downstream effects of SOCS2 inhibition on multiple disease phenotypes and immune disorders. The new SOCS2 ligands also offer attractive starting points for harnessing the ubiquitylation ability of the SOCS2 Cullin5 E3 ligase for targeted protein degradation.

Linossi et al. identified an important exosite on the SOCS2-SH2 domain which enhances the binding of pY ligands. In this account the binding of a non-phosphorylated peptide (F3) to the exosite greatly enhanced the binding affinity of canonical pY ligands; this peptide appears to bind on the opposite side of the classical pY binding pocket and induce a stabilisation of the SH2 domain, resulting in reduced dissociation of phosphorylated ligands. The authors proposed in this work that this exosite could potentially be exploited therapeutically, by enhancing SOCS2 suppression of inflammatory disease (69).

With respect to future drug design efforts, SOCS2 accounts for *ca.* 60% of the total number of structures of the SOCS family members, with all SOCS2 structures determined by X-ray crystallography. The heavy weighting of PDB entries towards SOCS2 may be attributed to the fact that significant structure-guided design efforts have been employed by the Ciulli laboratory, leading to many entries containing ligand-bound structures, and to the fact that the SOCS2-ElonginB-ElonginC (S_2_BC) complex is readily expressed and purified from *E. coli*, therefore, obtaining high-purity recombinant protein is not a limiting step in the structure determination workflow (14). This combination of ready access to protein for screening campaigns and a wealth of crystallographic information poises SOCS2 for further investigation.

## SOCS3

### SOCS3 exhibits unique binding conformations and directly inhibits JAK1, JAK2, and TYK2

**Figure 5:**
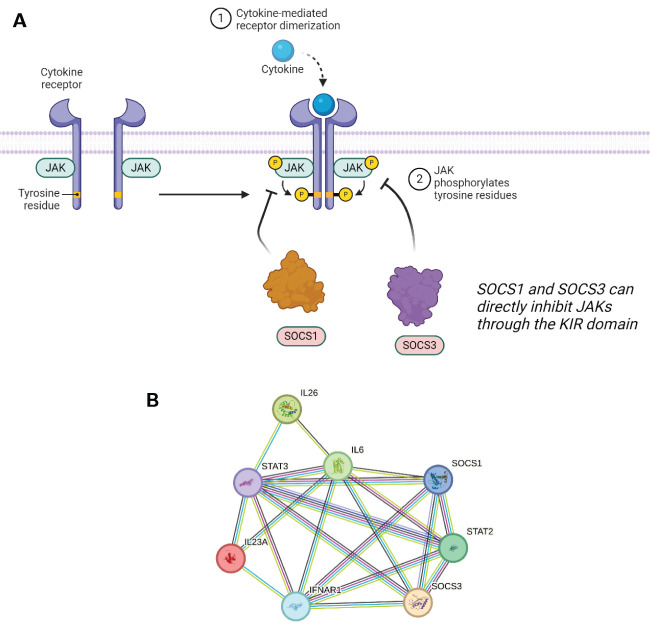
**(A)** SOCS1 and SOCS3 can directly inhibit JAK1/2 and JAK1/2/3 respectively, and/or induce proteasomal degradation by binding to pY residues. **(B)** STRING network showing the overlap in protein–protein interactions between SOCS1 and SOCS3. A legend explaining protein identity is available in the supporting information. In terms of known interactions, cyan-coloured strings are from curated databases and purple-coloured strings are experimentally determined. In terms of predicted interactions, green represents gene neighbourhood analyses, red are gene fusions events, and blue are from gene co-occurrence. The other remaining interactions represented in the STRING database include co-expression (black), protein homology (navy blue) and text-mining (olive).

SOCS3 shares structural similarity to SOCS1 in its KIR domain but also with CISH in that it contains a Proline-Glutamic Acid-Serine-Threonine (PEST) sequence. PEST motifs in SOCS3 were found to be crucial for the proteins ability to induce degradation - a study by Babon et al. showed that by removing the PEST sequence that laid between two secondary structures, there was an alteration in the intracellular degradation pathway. The PEST motif is located between the SH2 domain and SOCS box, two regions that are pivotal to how the proteins interact. Interestingly when PEST is removed, it was found that the main SOCS3 clearance mechanism is proteasomal degradation (70), and loss of PEST does not disrupt the SH2/pY binding interaction. SOCS3 contains an SH2 domain and so any phosphorylated tyrosine site is a potential substrate to bind to and tag for degradation (71). This statement is uniform across the SOCS protein family, even though SOCS1 and SOCS3 have a lower affinity for the E3 ligase machinery compared to the others. Instead, SOCS1 and SOCS3 bind directly to JAKs through their KIR, thereby inhibiting their kinase ability to phosphorylate downstream proteins (**Figure 5**).

**Figure 6:**
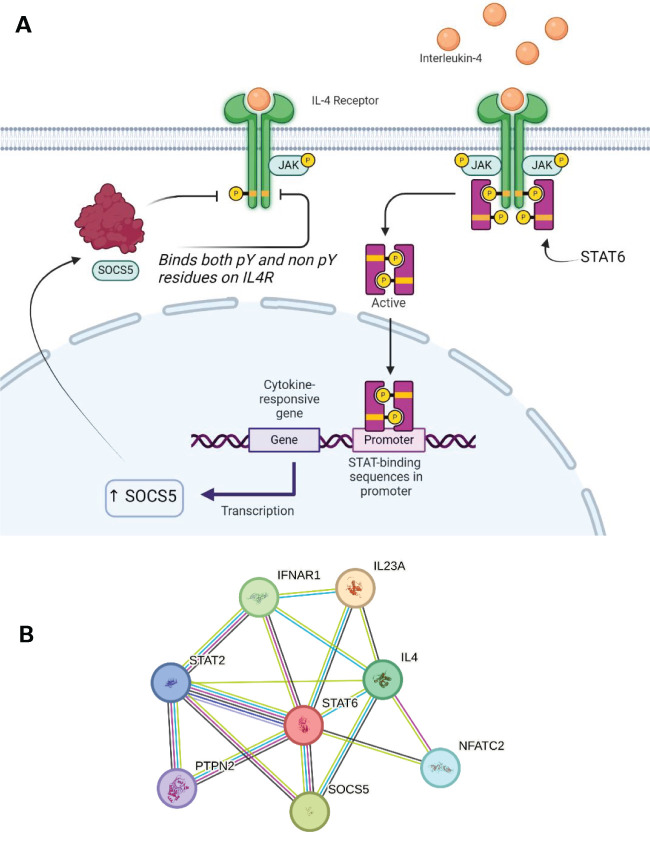
**(A)** SOCS5 expression can be triggered by IL4 signalling through JAK1/3 and STAT6. SOCS5 can inhibit this receptor through both phosphorylated and non-phosphorylated regions. **(B)** STRING network showing the overlap in protein-protein interactions between SOCS5 and SOCS2/5. A legend explaining protein identity is available in the supporting information. In terms of known interactions, cyan-coloured strings are from curated databases and purple-coloured strings are experimentally determined. In terms of predicted interactions, green represents gene neighbourhood analyses, red are gene fusions events, and blue are from gene co-occurrence. The other remaining interactions represented in the STRING database include co-expression (black), protein homology (navy blue) and text-mining (olive).

Out of the four JAKs, SOCS3 can successfully inhibit JAK 1, JAK 2, and TYK2. SOCS3 showed a unique binding conformation in its inhibition ability. It can simultaneously bind a JAK and a phosphorylated residue on the receptor tail. The generation of a ternary complex is specific to the combination of JAKs and STATs recruited which is dependent on which cytokine signalling system is activated in response to a specific stimulus. SOCS3 expression can be induced through IL-6, 12, 23, and granulocyte colony-stimulating factor (G-CSF) cytokine signalling. IL-6 cytokines are predominantly secreted by monocytes and do so in response to other inflammatory cytokines such as IL-11. The IL-6 family is a key activator of the STAT3 pathway which in combination with the gp130 receptor results in the expression of SOCS3 as well as other functional proteins. IL-6 binds to its transmembrane receptor which leads to the homodimerization of gp130 forming a complex with the receptor. In the case of SOCS3 and its role in inflammatory pathways, IL-6 signals through JAK3/STAT3. IL-6 is as a multifunctional cytokine with plethora of roles and physiological consequences including gene expression promoting hepatocyte synthesis, bone metabolism, and regulation of T cell differentiation (72). This signalling network is highlighted in in **Figure 5B** below, which showcases several key interactions, such as that of SOCS1 and SOCS3 with STAT2 and STAT3, and also with multiple interleukins. The overlap between this interaction network and that of SOCS5 is discussed further in reference to **Figure 6B**.

IL-6 signalling triggers the differentiation of Th17 cells which then go on to secrete a number of pro-inflammatory cytokines and so the immune response is amplified by multiple cytokines from the initial IL-6 cytokine. IL-6 signalling can promote T cell proliferation and boost the T-cell mediated immune response, making it particularly attractive for therapeutically modulating the adaptive immune response. SOCS3 has a contestable function in that it can act on this system to slow it down which proves useful in protecting our cells from an excessive inflammatory response (73).

SOCS3 will only inhibit STAT3 when its expression is stimulated via IL-6. This specificity is due to the direct interaction between SOCS3 and gp130 which allows the IL-6 signalling cascade to be targeted and not those triggered by other cytokines (74). IL-6 activates the STAT3 pathway in pancreatic cells which induces expression of SOCS3, which in turn regulate STAT3 activation by binding gp130. Lesina et al. observed that deletion of SOCS3 increased STAT3 phosphorylation as well as downstream development of pancreatic intraepithelial neoplasia (PanIN) throughout the pancreas. This suggested that SOCS3 was acting as a suppressor on KRAS, regulator of cell division protein, and limited the development and growth of pancreatic cancer lesions (75). SOCS3 has also been shown to act on a common autoimmune disease, rheumatoid arthritis. IL-6 signalling induced pro-inflammatory cytokine production which causes the severe inflammatory effect. SOCS3 is implicated in feedback loops within this system to reduce the production of these pro-inflammatory cytokines (76).

The therapeutic attraction for SOCS3 lies within its broad regulatory ability as well as its vital function in the inflammatory pathway, immune cell differentiation, and both bacterial and viral infection (76, 77). In relation to cancer drug therapy and SOCS3’s role as a suppressor of STAT3 activation, there has already been evidence shown in inhibiting growth of non-small lung cancer cells as well as overexpression of SOCS3 limiting the growth of malignant fibrous histiocytoma through inhibition of STAT3 and IL-6 production.

### Attenuating disease by silencing or inhibiting SOCS3

Knockdown of SOCS3 has been extensively studied in mouse models. SOCS3 can be strongly induced by a number of factors, including lipopolysaccharide and IL-10 (78–81). One study showed that mice with reduced SOCS3 expression in myeloid cells exhibit increased levels of pro-inflammatory cytokines, such as TNFα and IL-6 under LPS sepsis conditions, correlating with prolonged STAT3 activation (82). SOCS3^KD^ mice are conversely resistant to endotoxemia, which implies that SOCS3 blocks IL-6 mediated anti-inflammatory responses via STAT3 phosphorylation, resulting in a reduction of inflammatory cytokines (83). Yu et al. also identified that genetic deletion of SOCS3 in the T cells of mouse models were resistant to experimental autoimmune uveitis. Further analysis indicated that SOCS3 promotes the expansion of Th17/IFN-γ CD4 T cell populations, implicating SOCS3 in the progression of severe uveitis and framing it as a potential target for medicinal chemistry campaigns targeting uveitis and other autoimmune diseases (84).

In terms of efforts to drug SOCS3, Dumpati et al. described a structure-guided approach in 2016, treating SOCS3 as a therapeutic target for type 2 diabetes mellitus. Using a large virtual screen comprised of current diabetes treatments as well as general ligand libraries the authors identified a number of novel binders of SOCS3, primarily for the SH2 domain, which would enable the inhibition of SOCS3’s activity (85).

One further method of inhibiting the effects of SOCS3 is through microRNA therapy, through which SOCS3 can be downregulated (86). Li et al. have demonstrated that this SOCS3 inhibition can reduce apoptosis in certain pancreatic cells which enhancing the insulin secretion. Pedroso et al. concluded that selective inactivation of SOCS3, by antagonists or otherwise, can attenuate insulin resistance, and that SOCS3 inhibitors could be an attractive therapy for treating certain metabolic disorders. However, the authors also raised concerns that due to the essential role of SOCS3 in maintaining glucose homeostasis, it’s inactivation might present disadvantages under a number of physiological conditions (87). In a study on epithelial basal keratinocytes SOCS3^KO^resulted in severe skin inflammation (88), whereas SOCS3 overexpression in mouse models displayed characteristics of ‘chronic wounds’ as the result of prolonged inflammation.

**Figure 7:**
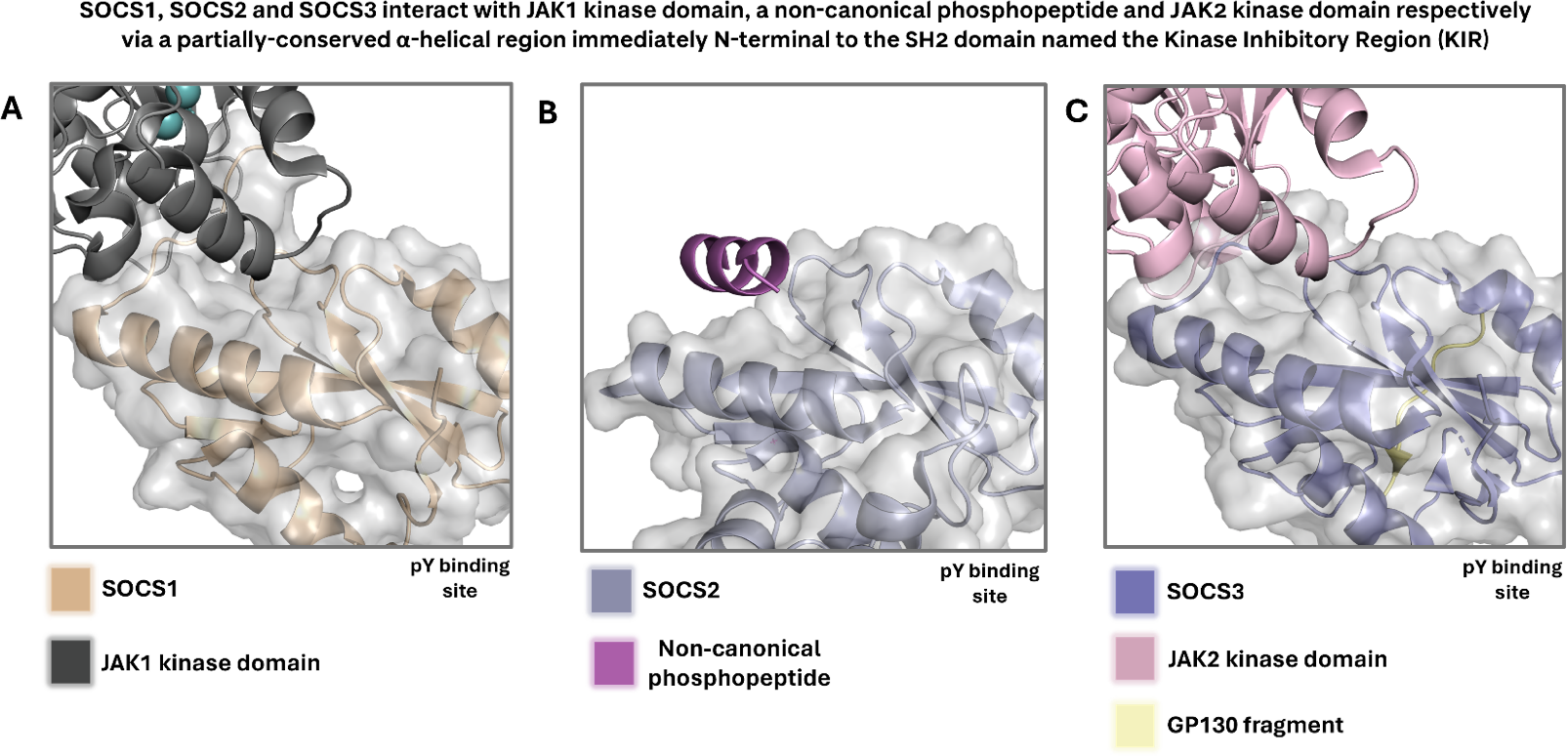
**(A)** Crystal structure highlighting the KIR of SOCS1 in complex with JAK1 kinase domain. Adapted from PDB 6C7Y. **(B)** Crystal structure of SOCS2, which is not known to contain a KIR but contains a partially conserved α-helical region, in complex with a non-canonical phosphopeptide. Adapted from PDB 7M6T. **(C)** Crystal structure highlighting the KIR of SOCS3 in complex with JAK2 kinase domain. Adapted from PDB 4GL9. SOCS1 and SOCS3 are highly homologous, and their KIR is partially conserved, indicating a correlation between sequence and structural conservation and mechanism of JAK inhibition. In all displayed crystal and solution NMR structures, waters and monomers likely derived from buffer components have been removed for clarity. In all cases, the surface is displayed for the SOCS protein only.

SOCS3 has been extensively studied by the Babon group, who have revealed several insights into the nature of protein-protein interactions of SOCS3 with the adaptor protein complex EloBC and scaffolding protein Cul5. A range of structures exist which give insights into the various mechanisms by which the SOCS family interact with phosphorylated native substrates, the E3 ligase machinery, and with the JAK kinases they inhibit. It is also clear from these structures that specific interaction motifs are involved in these various protein-protein interactions. A number of X-ray crystal structures and in-solution protein-observed NMR structures exist for SOCS3 (70, 89–91). A partially conserved α-helical region immediately N-terminal to the canonical SH2 domain (Kinase Inhibitory Domain, KIR) drives interaction with JAK kinase domains, as highlighted in PDB entries for SOCS1 and SOCS3 with JAK1 and JAK2 kinase domains respectively (**Figure 7**) (20, 22, 92).

## SOCS4

### SOCS4 fulfils complex roles in EGF signalling

SOCS4 contains a considerably longer N-terminal domain in comparison to other SOCS proteins whilst sharing a distinctive N-terminal conserved region with SOCS5. The function of this region is yet to be determined. A study undertook bioinformatic analysis which indicated that this domain is largely disordered with a more orderly and structured *ca.* 70 residue region. The N-terminal of SOCS4 and SOCS5 have been shown to plays minor role in the mediating epidermal growth factor (EGF) signalling through regulation of the EGF receptor (EGFR).

**Figure 8:**
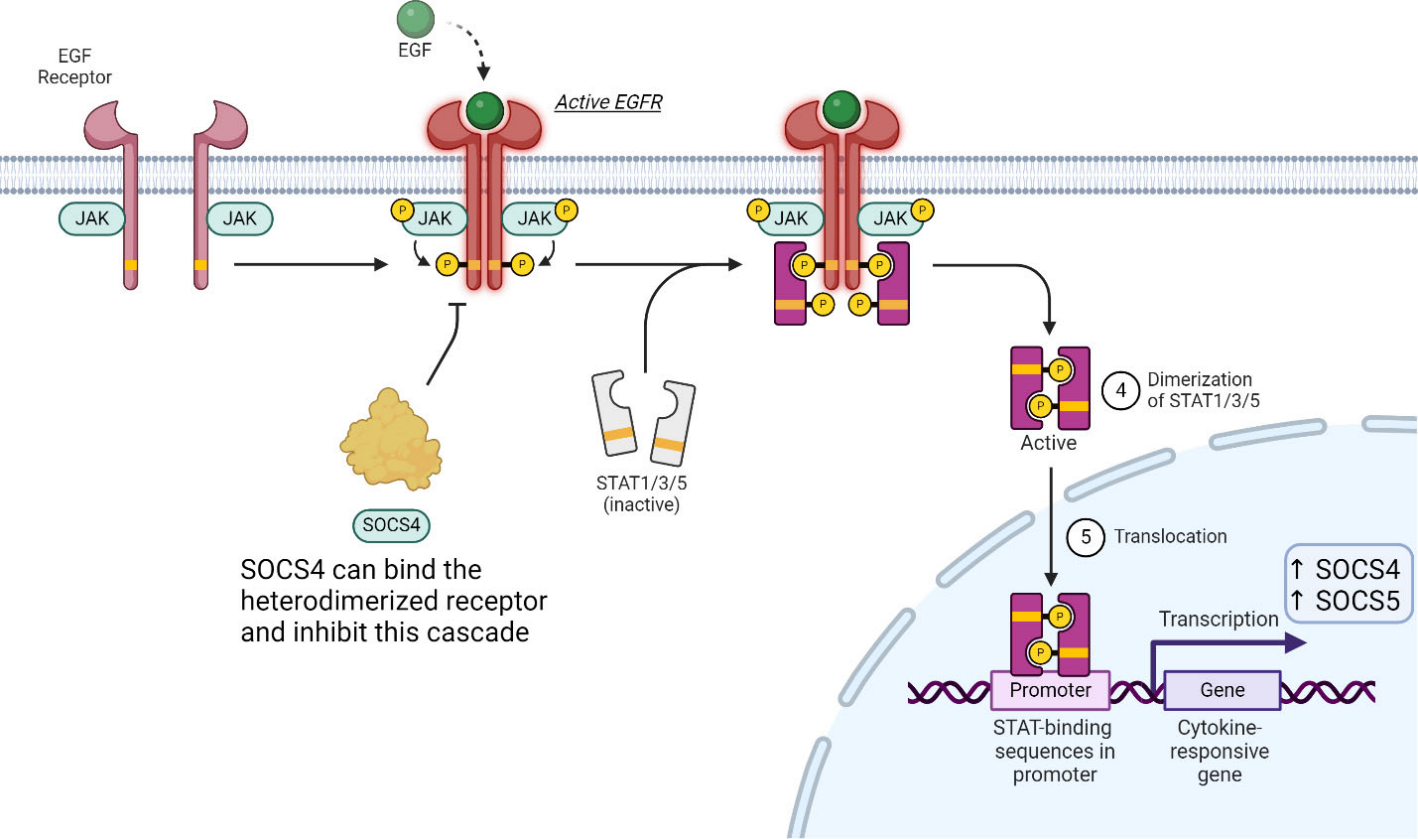
SOCS proteins can play a minor role of the JAK-STAT pathway in EGFR signalling. Binding of EGF induces heterodimerization of the receptor, which in turn activates multiple STAT proteins. SOCS4 can compete for pY resides, while SOCS5 (not shown) can bind non-phosphorylated tyrosine residues to affect this signalling cascade.

EGFR is known to be regulated by multiple STAT pathways. STAT1, 3 and 5 are all regulators of EGF signalling and activation of each triggers a different downstream response. EGF production can be stimulated by testosterone and the molecule itself can be found in platelets, urine, blood plasma, and saliva. There are multiple cytokines which can activate EGFR signalling and the outcome is dependent on the stimulatory ligand (93). Upon EGF binding to the monomeric receptor, conformational changes drive heterodimerization of the receptor subunits. Once in this activated form, downstream phosphorylation and therefor activation of STATs results in their translocation to the nucleus where binding to specific regulatory sequences results in the expression of SOCS4 (**Figure 8**).

Abnormal regulation of EGF signalling and EGFR expression plays an important role in oncogenesis (94). This identified EGFR as a target for expanding druggable receptors involved in cytokine and small molecule signalling pathways. SOCS4 and SOCS5 are the only SOCS proteins which have been shown to notably reduce the expression of EGFR yet there is still uncertainty in SOCS4’s direct function on the EGF signalling pathway. SOCS4 specific role in immune signalling has also yet to be identified. Studies were done using SOCS4^KO^ mice and results demonstrated that lacking SOCS4 resulted in rapid infection with a H1N1 influenza as well as being more susceptible to other pathogenic infections. This suggests SOCS4 may have a role in the regulation of pro-inflammatory cytokines and chemokines but more work in this area would be required to further conclude. (95).

Upon EGF stimulating the dimerization of EGFR, phosphorylated tyrosine’s act as docking sites for SOCS4 to bind and tag the receptor for proteasomal degradation. Although SOCS4 can act to reduce cellular EGFR levels, knockout studies were performed, and immune cell populations were monitored (95). There were no noticeable fluctuations in these populations and thus the mechanism of SOCS4 action remains largely unsolved. Research on SOCS4 to date does not provide a physiological role nor a therapeutic attractiveness for the protein as a regulator of the immune system despite its broad expression.

### SOCS4 knockout causes hypersusceptibility to influenza and is indicated in autoimmune disease

Feng et al. have conducted significant studies on the effects of SOCS4^KD^, and focused on investigating the mechanistic implications of both SOCS3 and SOCS4 expression levels on wound healing. The authors developed a SOCS4-deficient mouse model, and identified SOCS4’s crucial role in the inflammatory response to pathogen infection, namely influenza (96). In this same account, the authors also illuminated a previously undiscovered function of SOCS4 in modulating TCR signalling. Feng and co-workers proposed that SOCS4 likely plays an important role in wound healing, as its also interacts and regulated signalling and transcription factors such as HIF-1α (96). Conversely, Kedzierski et al. showed that SOCS4 is actually dispensable for a recall response in influenza A infection, while still crucial in the primary immune response. SOCS4-deficient mice are hypersusceptible to primary infection in influenza A and exhibit highly dysregulated pulmonary chemokine production, however the authors discovered that SOCS4 was not required for CD8^+^ T cell memory generation, and furthermore was not required to recall those cells in response to a secondary influenza infection. Despite an impaired initial immune response, SOCS4 was not found essential in preventing secondary infection with influenza (97).

SOCS4 mutations have been previously indicated in autoimmune disease. A clinical report from Arts et al. sought to understand a familial autoimmune disorder and elucidate the genetic components at play. The authors performed whole-exome sequencing on patients and identified a genetically-transmittable missense mutation in the SOCS4 gene as a likely culprit for the immune response dysregulation. Arts et al. concluded that a single mutation in the SOCS4, T266M, leads to impaired SOCS4 which insufficiently modulates signalling by EGFR and presents impaired STAT3 inhibition. The dysregulation of EGFR signalling results in excessive IL-6 production, which likely underpins this familial autoimmune disorder. (98).

With respect to ligand design and druggability, SOCS4 has generally undergone less investigation than its family members – a trend which continues through to SOCS7. However, some key structural insights have been published by Bullock et al. on the SOCS4/EloB/EloC complex, which revealed a distinct SOCS box interface (23). The authors previously identified a key evolutionary divergence between the SOCS box of SOCS4-7 and the earlier family members (23). While Bullock and co-workers highlighted a number of key structural differences in the binding domains of SOCS4 compared to its family members, and provided the basis for structure-guided drug design through this publication, there has been little to no development in inhibitors for SOCS4 or indeed the use of the SOCS4/EloB/C complex for targeted protein degradation.

## SOCS5

### SOCS5 uniquely inhibits JAK-STAT through binding to non-phosphorylated residues

SOCS5 expression is constitutive in both T cell and B cells yet the differentiation and development of both cell types is unaffected in the absence of SOCS5. This suppressor has relatively high expression in lymphoid organs such as the spleen and lymph nodes which suggests an immune related role. There has been minimal investigation into the function of SOCS5 but a past study did conclude a correlation in the reduction of IL-4R with a cytoplasmic presence of SOCS5 (99).

The IL-4 cytokine has an important role in the immune response and is ubiquitously expressed on both innate and adaptive immune cells. Its role in regulating inflammation and antibody production has made it a previous target for inhibition therapies to counteract diseases. The secretion of this pleiotropic cytokine is both induced and increased through multiple extracellular stimuli (100). For example, mast cells, eosinophils and basophils release IL-4 in response to ligation of allergens to specific surface-bound immunoglobulins (101). IL-4 signals through JAK1/3 and STAT6 (**Figure 6**). The combination of these intracellular proteins determines the outcome of downstream signalling. IL-4 signalling plays a key role in mediating Th2 cell development and the binding of SOCS5 to a region on the IL-4R results in the inhibition of IL-4 signalling (**Figure 6**). The downstream effects of this SOCS5 preferential interaction with the IL-4R was seen to affect the functionality of the IL-4R ability to activate STAT6. Interestingly, SOCS5 is the only known member of the family which can bind and inhibit a JAK-STAT pathway through binding to non-phosphorylated tyrosine residues. The mechanism of action is not fully understood but SOCS5 binding is thought to reduce the JAK1 association (27).

The wider immune system implications of IL-4R inhibition (as a possible result of SOCS5’s activity) have been shown to reduce Th2 development in naïve T cells. The Th1 response produces proinflammatories such as IFN-γ whilst Th2 cells function through activating antibody-mediated response. This inhibition was reversed when a large excess of IL-4 was added. There is a possibility that other SOCS proteins play a role in this IL-4-mediated STAT6 inhibition due to the marginal inhibition activity of SOCS5 alone. Recently, studies have investigated CISH and its influence in IL-4 signalling, although SOCS5 still may play a role in regulating the Th1 and Th2 balance through mediating the differentiation progression (35). Many of these protein-protein interactions are highlighted in the STRING network in **Figure 6B**. In particular, the overlap of this network with **Figure 5B** shows common interactions with Interleukin-23 subunit alpha (IL23A) and interferon-alpha/beta receptor alpha chain (IFNAR1) amongst SOCS1/3/5. Furthermore, in the context of cell differentiation, nuclear factor of activated T cell cytoplasmic 2 (NFATC2) is implicated in the SOCS5 interaction network.

### Decreased SOCS5 expression correlates with COPD in patients, and increasing susceptibility to infection

SOCS5^-/-^ mice do not have noticeable differences in their CD4^+^/CD8^+^ ratios (102). However, these knockout models are increasingly susceptible to infection with influenza A and exhibit increased levels of pro-inflammatory cytokines (103). These knockout mice lost significantly more body weight upon day 3 of infection when compared to wild-type, and an increased pulmonary viral load was identified. Kedzierski et al. noted that elevated influenza A levels were present in SOCS5-deficient lungs from day 1 of infection, suggesting that these mice had an impaired innate ability to resist early viral replication. Patients with chronic obstructive pulmonary disease (COPD) have been found to have decreased SOCS5 levels, which underpins heightened levels of IL-1β and TNFα (103). An account from Sharma et al. sought to elucidate the regulatory roles of SOCS5 in leukaemia and cellular signalling and found that genetic silencing of SOCS5 induces JAK-STAT signalling activation, and negatively regulates some interleukins (104). The authors postulated that SOCS5 inactivation accelerate leukaemia burden and progression of the disease.

Brender et al. compared SOCS5 in the context of innate immunity and identified key differences when compared to other protein family members. While SOCS1 and SOCS3 are heavily implicated in LPS and CpG responses, their SOCS5^-/-^ B cells responded normally to stimulation by these bacterial products, indicating that SOCS5 is not essential for innate immune response, and concluded that while it is expressed in primary B and T lymphoid cells, it is dispensable in their function (102).

Emerging evidence suggests that SOCS5 and its dysregulation play a role in disorders such as autoimmune uveoretinitis, multiple sclerosis, and type 1 diabetes (105–107). Further investigation of inhibitors and genetic deletion of SOCS5 will illuminate how this SOCS family member regulates immune responses.

## SOCS6

### SOCS6 feedback inhibition provides antileukemic effects

SOCS6 and SOCS7 share structural homology both with a similar length N-terminal region. The functionality of SOCS6 as an inhibitor has been predominantly associated with the regulation of haematopoiesis and the FLT3 transmembrane protein. Fms-like tyrosine kinase 3 (FLT3) is a class III receptor tyrosine kinase that transmits extracellular signals into the cell through signal transduction, following stimulation by the FLT3 cytokine. Ligand binding leads to the dimerization of the receptor triggering intrinsic tyrosine kinase activity, and this activated FLT3 pathway results in phosphorylation of STAT5 through its docking at phosphotyrosine sites. Once this pSTAT5 dimerises and translocate to the nucleus to bind to regulatory sequences, SOCS6 expression is induced. Cytoplasmic SOCS6 is then able to feedback on this same system to control the output of FLT3 signalling (108).

SOCS6 inhibition of FLT3 receptor involves the SH2 domain of SOCS6 binding to a specific phosphorylated tyrosine site. This action not only out competes STAT5 for the docking site, but also results in the ubiquitination and receptor internalisation. A decrease in FLT3 signalling because of SOCS6 feedback inhibition can have a plethora of antileukemic affects and as such, FLT3 is an attractive therapeutic target for better understanding and control Acute Myeloid Leukaemia diseases (109).

### SOCS6 is primarily involved in regulating kinase signalling

Knockout of SOCS6 appears to have very little effect on organism phenotype, other than slightly decreased growth (110). Unlike the other family members, SOCS6 is primarily involved in negative regulation of receptor tyrosine kinase signalling through ubiquitin-dependent degradation inducing apoptosis by targeting mitochondrial proteins (111). Kabir et al. note in their 2014 account that both SOCS6^KO^ and indeed SOCS7^KO^ mice are viable and mostly normal, except for a *ca.* 10% weight reduction. Although SOCS6 is ubiquitously expressed across hematopoietic compartments, SOCS6^KO^ mice did not exhibit any changes in spleen or bone marrow. Furthermore, while SOCS6 associates with IRS1, 2, and 4 to regulate insulin signalling, SOCS6^KO^ mice displayed normal responses to insulin and demonstrated normal metabolism of glucose – it has been postulated that SOCS7 can ‘step-in’ in SOCS6^KO^ models to compensate (111–113).

SOCS6 does not mediate signalling via classical SOCS targets such as growth hormone or prolactin, and it is likely that SOCS6 is not actually involved in maintaining the balance of cytokine signalling, rendering it somewhat less relevant to the regulation of the immune system. SOCS6 has not been observed to have a suppressive function in lymphocytes stimulated by interleukins, and in a study from Li et al. it did not inhibit phosphorylation of STAT1 through STAT6 (112).

## SOCS7

Structurally, SOCS7 is very alike to SOCS5 and SOCS6 in its N-terminal length. SOCS7 was found to be the only family member which contains a putative nuclear localisation signal which is responsible for anchoring the protein to be imported into the nuclei. Although structurally similar to its previous family members, SOCS7 has not been well characterised in its negative regulation of cytokine signalling.

SOCS7 is known to be expressed and act on the IGF-1 signalling pathway. Stimulation of the IGF-1 receptor is through ligand binding of GH which is traditionally synthesised and secreted by cells in the pituitary gland. It has been proposed that SOCS7 is implicated in a JAK2/STAT5 signalling cascade (114). Inhibition by SOCS7 is achieved by subjecting the receptor to proteasomal degradation via recruitment of the E3 ubiquitin ligase scaffold. SOCS7 has also been shown to negatively regulate this system through binding to phosphorylated tyrosine residues via its SH2 domain to out compete STAT protein activation. The downstream effect of this feedback could disrupt metabolism and homeostasis leading to insulin resistance.

### SOCS7 is heavily implicated in allergic inflammation, and onset of cytokine storm

SOCS7 exhibits high expression levels in the brain, and similar to SOCS6, SOCS7^KO^ mice are on average *ca.* 10% smaller than their wild-type littermates. SOCS7 appears to be crucial in the function of neuronal cells with SOCS7^KO^ mice exhibiting hydrocephalic symptoms such as ventricular system dilation and cranial distortion, with 50% of these mice dying within 15 weeks of age (115). SOCS7 has been indicated by Banks et al. as a modulator of insulin signalling and glucose homeostasis, as seen with other SOCS members. The authors identified that SOCS7^-/-^ mice display prolonged hypoglycaemia and lower glucose levels during insulin tolerance tests – many of the defects in SOCS7-deficient mice can be explained by enhanced inclusion action in SOCS7^KO^ cells and increased IRS levels (116).

SOCS7 has been heavily implicated in severe skin disease and allergic inflammation. An account from Knisz and co-workers found that SOCS7-deficient mice suffered from severe cutaneous disease with increased mast cell activation (117). In a hydrocephaly-resistant SOCS7^-/-^ mouse model, severe cutaneous disease was evident in 50% of the population after 16 months of age and furthermore the SOCS7^-/-^ mice exhibited significantly increased mast cell numbers, which were hyperactive to stimulus with IgE. This hyperactive response manifested with increased proinflammatory cytokine production, further implementing SOCS7 in immune regulation (27, 117). While the roles of SOCS7 in human disease and immune dysregulation has not been thoroughly investigated, one account form Sasi et al. has indicated a possible tumour-suppressing role of this SOCS family member, with high SOCS7 expression correlating with increased survival. In this account, Sasi et al. uncovered a regulatory role of SOCS7 in ER-positive and ER-negative breast cancer cells, by acting on insulin-like growth factor (IGF-1)-induced functions (118).

Loss of SOCS7 has been implicated in cytokine storm onset, in which serious damage to tissue and organs results in a large amount of cytokines being expressed in a short time – cytokine storm is one cause of hepatocyte necrosis in the development of acute liver failure (ALF). Fu et al. demonstrated that protein tyrosine phosphatase non-receptor type 14 (PTPN14) interacts with SOCS7 and induces the proteasomal degradation of SOCS7 and aggravates inflammation in ALF (119). This inhibition and proteasomal destruction of SOCS7 severely impacts SOCS7 levels and weakens its mediating effects on the inflammatory responses. In this account the authors established the PTPN14-SOCS7-NFkB axis as a network in inflammatory regulation and offer it as a potential drug target for ALF. (119).

A recent account from Cornebois et al. disclosed the versatility of SOCS7 as an E3 ligase for targeted protein degradation (120). The authors highlight a protein-based degrader method in which an anti-tag intracellular antibody is fused to a series of E3 ligases to enable efficient screening for degradation of a tagged target protein. SOCS7 was identified from the screen as a potent biodegrader, which initiated degradation of the target protein across multiple cell lines. Furthermore, the authors disclosed a SOCS7-based KRAS degrader – a major oncology target which has been the subject of several medicinal chemistry campaigns. The authors utilised a truncated protein to replace the original substrate binding domain for these SOCS7-based biodegraders, which underpins the unique potential of the SOCS domain for therapeutic development.

In a similar context, a preprint from Magdaleno et al. further investigated the interactome of the SOCS domain within the context of idiopathic pulmonary fibrosis (IPF) – a fatal disease which is characterised by lung scarring and extracellular matrix (ECM) protein accumulation, resulting in damaged lung function. The authors highlight that the SOCS domain targets VHL for proteasomal degradation, and that the conserved SOCS domain can disrupt ECM fibrils associated with fibrotic lung myofibroblasts. Magdaleno et al. identified that upon fibroblast differentiation and subsequent SOCS protein transduction, reduced levels of the contractile myofibroblast marker Alpha Smooth Muscle Actin (α-SMA) were observed. The authors expanded on these discoveries by delivering the SOCS domain in the fibrotic phase of lung fibrosis through an adenoviral vector and found that treated mice presented significantly reduced collagen accumulation in diseased lungs. Magdaleno et al. proposed that the SOCS domain has a previously unidentified function in potentially disrupting pathological matrix deposition in lung fibroblasts (121).

## Conclusions and outlook

The SOCS family holds a privileged role in the regulation of the immune system, with a wide array of important signalling functions of complex framework. Silencing experiments have proven the inextricable link between this important protein family and disease, susceptibility to infection, and allergic response. Their roles extend from the regulation of growth hormone responses and prolactin signalling, to balancing insulin responses and a multitude of interleukins.

While drug design approaches to small molecule inhibitors have been somewhat limited, the SOCS proteins offer a valuable target family for the regulation of the immune system at multiple levels and small-molecule chemical probes are beginning to emerge. To date most medicinal chemistry efforts have centred on virtual screens for the SOCS family members, with the exception of SOCS2 for which a structure-guided campaign led to the development of a covalent inhibitor qualified as SOCS2 “handle”. We have examined the intricate roles of SOCS proteins in a signalling context and identified how drugging and/or regulating these proteins could offer therapeutic advantages in terms of immunological diseases and beyond. Furthermore, structural characterisation of PPIs with known substrates exists for several SOCS family members. The importance of such data for enabling medicinal chemistry campaigns similar to that described for SOCS2 cannot be understated. We have highlighted key crystallographic information, already present in the literature, which facilitates further drug discovery campaigns. Throughout the present review, we have also alluded to the SOCS proteins as a possible family for expanding the toolbox for targeted protein degradation, with SOCS7 recently being utilised for this new modality.

Continued research will further elucidate the structural relationships of these proteins and their interactomes, illuminating the vast network of cytokine homeostasis, and will usher in a new age of therapeutic interest, as drug targets in their own right, or E3 ligases hijackable for targeted protein degradation.

## Supplementary material

Supplementary Table 1 Summary of PDB entries for the SOCS family (ordered by SOCS family member).
