# Supplementary material for: Unravelling the druggability and immunological roles of the SOCS-family proteins
Source: Front Immunol. 2024 Nov 29;15:1449397. doi: 10.3389/fimmu.2024.1449397 (PMC11638205; doi:10.3389/fimmu.2024.1449397)
Supplement: Supplementary Table 1 — Summary of PDB entries for the SOCS family (ordered by SOCS family member). [file DataSheet1.docx]

**Supporting Information**

| SOCS Family Member | Structure Determination Method | Resolution | PDB Entry | Comments |
| --- | --- | --- | --- | --- |
| SOCS1 | X-ray | 2.50 Å | 6C7Y | SOCS1 in complex with JAK1 kinase domain |
| SOCS1 | X-ray | 3.11 Å | 6C5X | SOCS1:ElonginB:ElonginC complex with a GP130 phosphopeptide-fragment |
| SOCS2 | X-ray | 2.8 Å | 6I5J | SOCS2:ElonginB:ElonginC in complex with growth hormone receptor peptide |
| SOCS2 | X-ray | 1.90 Å | 2C9W | SOCS2:ElonginB:ElonginC complex |
| SOCS2 | X-ray | 2.99 Å | 5BO4 | SOCS2:ElonginB:ElonginC complex |
| SOCS2 | X-ray | 2.69 Å | 6I4X | SOCS2:ElonginB:ElonginC complex with erythropoietin receptor peptide |
| SOCS2 | X-ray | 1.98 Å | 6I5N | SOCS2:ElonginB:ElonginC in complex with growth hormone receptor peptide |
| SOCS2 | X-ray | 3.19 Å | 7M6T | SOCS2:ElonginB:ElonginC bound to a non-canonical peptide that enhances phospho-peptide binding |
| SOCS2 | X-ray | 1.79 Å | 7ZLM | SOCS2:ElonginB:ElonginC in complex with MN551 (optimized covalent SH2 domain binder) |
| SOCS2 | X-ray | 2.6 Å | 7ZLN | SOCS2:ElonginB:ElonginC in complex with an SH2 domain binder |
| SOCS2 | X-ray | 2.22 Å | 7ZLO | SOCS2:ElonginB:ElonginC in complex with an SH2 domain binder |
| SOCS2 | X-ray | 1.94 Å | 7ZLP | SOCS2:ElonginB:ElonginC in complex with an SH2 domain binder |
| SOCS2 | X-ray | 2.01 Å | 7ZLR | SOCS2:ElonginB:ElonginC in complex with an SH2 domain binder |
| SOCS2 | X-ray | 1.92 Å | 7ZLS | SOCS2:ElonginB:ElonginC in complex with an SH2 domain binder |
| SOCS2 | X-ray | 3 Å | 4JGH | SOCS2:ElonginB:ElonginC:Cullin5NTD complex |
| SOCS3 | Protein-observed NMR | - | 2JZ3 | SOCS3:ElonginB:ElonginC complex – in solution structure |
| SOCS3 | Protein-observed NMR | - | 2BBU | Mouse SOCS3 in complex with a GP130 phosphopeptide – in solution structure |
| SOCS3 | X-ray | 2 Å | 2HMH | SOCS3 in complex with GP130(pTyr757) phosphopeptide |
| SOCS3 | X-ray | 3.9 Å | 4GL9 | SOCS3 in complex with JAK2 kinase domain and fragment of GP130 intracellular domain |
| SOCS4 | X-ray | 2.55 Å | 2IZV | SOCS4:ElonginB:ElonginC complex |
| SOCS5 | Protein-observed NMR | - | 2N34 | Disordered N-terminal domain – in solution structure |
| SOCS6 | X-ray | 1.45 Å | 2VIF | SOCS6 SH2 domain in complex with a c-KIT phosphopeptide |

**Table S1:** Summary of PDB entries for the SOCS family (ordered by SOCS family member).

**STRING Database Legend**

In terms of known interactions, cyan-coloured strings are from curated databases and purple-coloured strings are experimentally determined. In terms of predicted interactions, green represents gene neighbourhood analyses, red are gene fusions events, and blue are from gene co-occurrence. The other remaining interactions represented in the STRING database include co-expression (black), protein homology (navy blue) and text-mining (olive).

In Figure 4 (B), the following proteins are indicated in the interaction web between SOCS1 and SOCS3:

STAT2 – signal transducer and activator of transcription 2

STAT3 – signal transducer and activator of transcription 3

IL6 – Interleukin-6

IL26 – Interleukin-26

IL23A – Interleukin-23 subunit alpha

IFNAR1 – Interferon-alpha/beta receptor alpha chain

In Figure 6 (B), the following proteins are indicated in the interaction web between SOCS1 and SOCS3:

STAT2 – signal transducer and activator of transcription 2

STAT6 – signal transducer and activator of transcription 6

IL4 – Interleukin-4

IL23A – Interleukin-23 subunit alpha

IFNAR1 – Interferon-alpha/beta receptor alpha chain

PTPN2 – Tyrosine-protein phosphatase non-receptor type 2

NFATC2 – Nuclear Factor Of Activated T Cells, cytoplasmic 2
